# Nrf2 and Oxidative Stress: A General Overview of Mechanisms and Implications in Human Disease

**DOI:** 10.3390/antiox11122345

**Published:** 2022-11-27

**Authors:** Vy Ngo, Martin L. Duennwald

**Affiliations:** 1 Department of Pathology and Laboratory Medicine, Schulich School of Medicine and Dentistry, University of Western Ontario, London, ON N6A5C1, Canada; 2 Department of Anatomy and Cell Biology, Schulich School of Medicine and Dentistry, University of Western Ontario, London, ON N6A5C1, Canada

**Keywords:** Nrf2, oxidative stress, antioxidant, antioxidant response

## Abstract

Organisms are continually exposed to exogenous and endogenous sources of reactive oxygen species (ROS) and other oxidants that have both beneficial and deleterious effects on the cell. ROS have important roles in a wide range of physiological processes; however, high ROS levels are associated with oxidative stress and disease progression. Oxidative stress has been implicated in nearly all major human diseases, from neurogenerative diseases and neuropsychiatric disorders to cardiovascular disease, diabetes, and cancer. Antioxidant defence systems have evolved as a means of protection against oxidative stress, with the transcription factor Nrf2 as the key regulator. Nrf2 is responsible for regulating an extensive panel of antioxidant enzymes involved in the detoxification and elimination of oxidative stress and has been extensively studied in the disease contexts. This review aims to provide the reader with a general overview of oxidative stress and Nrf2, including basic mechanisms of Nrf2 activation and regulation, and implications in various major human diseases.

## 1. Oxidative Stress

### 1.1. Reactive Oxygen Species

Free radicals are unstable atoms, ions, or molecules containing one or more unpaired electrons in the outermost electron shell. An unpaired valence electron is unstable and highly reactive. To attain stability, free radicals attack and acquire electrons from other compounds or molecules within their proximity. The attacked entity loses an electron to become oxidized and becomes a free radical itself, thereby initiating a chain reaction that can result in cellular damage [1]. ROS and reactive nitrogen species (RNS) are unstable molecules containing oxygen and/or nitrogen and include both free radical and non-radical species. The oxygen molecule (O_2_••) is a weak free radical itself due to the presence of two unpaired electrons in its valence shell; however, it is less reactive than other oxygen species due to the parallel spin of its electrons [2]. Major ROS and RNS are listed in Table 1.

RNS is a family of nitrogen moieties associated with oxygen. They are produced when nitric oxide (•NO) reacts with oxygen species. For example, nitric oxide can react with superoxide (O_2_•^−^) to form peroxynitrite (ONOO^−^):

•NO + O_2_•^−^→ONOO^−^ (1)

Peroxynitrite is very reactive and readily attacks lipid molecules, resulting in lipid peroxidation and lipoprotein oxidation [3]. However, like ROS, low levels of RNS have important roles in physiological processes. For example, nitric oxide produced by nitric oxide synthase (NOS) regulates blood vessel dilation and is involved in synaptic transmission in the brain [4,5]. On the other hand, high levels of RNS result in nitrosative stress, macromolecule damage, and activation of transcription factors NF-_Κ_B and activator protein 1 (AP-1) involved in inflammation and other pathological pathways [6,7]. RNS and ROS often act together to cause cellular damage [8].

**Figure 1:**
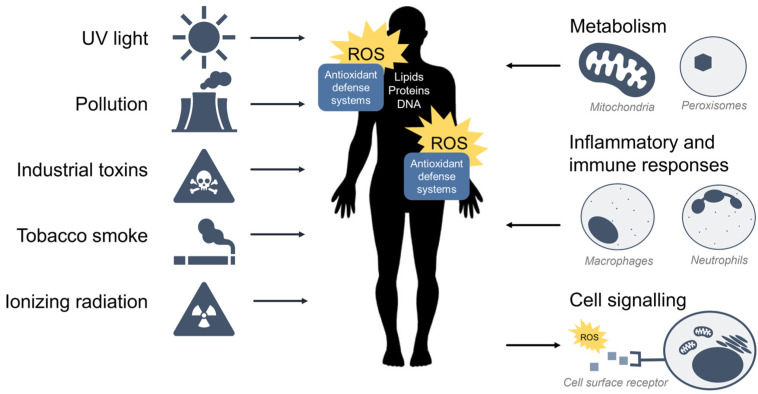
Sources of exogenous and endogenous ROS. ROS can come from toxic exogenous sources in the environment, or be produced as by-products of normal cell metabolism, inflammation, and immunity. ROS may also function as secondary messengers within cell signalling pathways.

ROS are oxidants (i.e., a molecule that removes electrons from other molecules) predominantly produced as byproducts of cellular metabolism and biochemical processes within the cell. Mitochondria are a primary source of ROS produced by aerobic respiration [9,10,11,12], where the reduction of molecular oxygen in the electron transport chain results in the leaking of superoxide radicals which are readily detoxified to hydrogen peroxide (H_2_O_2_) by antioxidant enzymes such as catalase and glutathione peroxidase. Hydrogen peroxide may react with transition metals such as iron (Fe^2+^) to produce hydroxyl radicals via the Fenton reaction to further produce hydroxyl radicals (•OH) which are highly reactive toward all components of DNA molecules as well as lipids [13]. Peroxisomes also generate ROS from aerobic metabolism [14], and phagocytic neutrophils and macrophages produce ROS to eliminate invading pathogens [15]. At low to moderate levels, ROS plays an important role in normal cellular processes, serving as secondary messengers in intracellular signalling cascades that mediate cell growth, autophagy, inflammatory and immune function, and contribute to overall redox regulation [16,17]. However, both radical and non-radical ROS can be powerful oxidants that are detrimental to the cell upon high or chronic exposure. Toxic exogenous sources of ROS include pollution, tobacco smoke, alcohol, ozone, environmental and industrial toxins, and radiation [1]. Due to their reactive nature, ROS production and elimination must be strictly regulated by the cell. Figure 1 summarizes the major sources of exogenous and endogenous ROS and their outcomes in the cell.

### 1.2. Oxidative Stress

Extensive or prolonged exposure to ROS results in oxidative stress, a deleterious process that damages lipids, proteins, and nucleic acids in the cell, thereby inhibiting their normal function [2]. In this scenario, there is an imbalance between the production of ROS and cellular defence mechanisms against oxidative stress, i.e., the antioxidant response. Chronic oxidative stress and the resultant oxidative damage have been implicated in many human diseases including cardiovascular disease, neurodegenerative diseases, diabetes, cancer, and the aging process [18,19,20,21,22].

The consequence of ROS or oxidants and the extent of oxidative stress depends on the strength, duration, and context of exposure. In response to oxidative stress, cells typically undergo cell cycle arrest and enter the G_0_ phase (i.e., a quiescent, non-dividing stage) due to activation of the p53-regulated cyclin-dependent kinase inhibitor p21, which halts cell cycle progression and inhibits DNA synthesis [23,24]. ROS can also trigger the p53 and p21-mediated dephosphorylation and activation of the tumour-suppressor retinoblastoma protein (Rb) resulting in further inhibition of cell cycle progression [25]. It is interesting to note that p21 is also involved in the regulation of the antioxidant response through its binding to the antioxidant transcription factor, Nrf2 [26] (to be discussed in Section 2.6). Depending on the nature of the exposure, cells can activate adaptive cell survival pathways; however, chronic exposure or excessively high levels of ROS may result in the induction of maladaptive autophagic or apoptotic pathways [27,28].

To preserve the delicate balance between the beneficial and harmful effects of ROS, living organisms have evolved cellular defence mechanisms against oxidative stress to maintain redox homeostasis. Alterations in redox status can lead to the transcriptional activation of pathways and enzymes involved in the detoxification, transport, and elimination of ROS. For further reading on oxidative stress, the reader is encouraged to refer to the comprehensive review by Sies et al., (2017) [29].

### 1.3. Antioxidant Response Enzymes

Complex antioxidant defense systems have evolved to protect cells and tissue against oxidative stress. Halliwell and Gutteridge have defined antioxidants as “any substance that, when present in low concentrations compared to that of an oxidizable substrate, significantly delays or inhibits the oxidation of that substrate” [30]. Key antioxidant defenses include (1) antioxidants that directly scavenge ROS, such as glutathione, vitamin C, and vitamin E, and (2) antioxidant enzymes including superoxide dismutase, catalase, and glutathione peroxidase.

**Superoxide dismutases (SOD)** are a class of enzymes found within the cytosol and mitochondria of nearly all aerobic cells and contain metal ion cofactors such as copper, zinc, manganese, or iron. SOD isoenzymes include Cu/Zn-SOD (SOD1), Mn-SOD (SOD2), and extracellular (EC) SOD (SOD3) [31,32]. SODs are responsible for the dismutation (simultaneous oxidation and reduction) and breakdown of superoxide radicals into molecular oxygen and hydrogen peroxide

SOD
2O_2_•^−^ + 2H^+^→O_2_ + H_2_O_2_ (2)

Molecular oxygen and hydrogen peroxide are weak oxidants that are relatively stable; however, hydrogen peroxide can be converted into extremely reactive hydroxyl radicals and must therefore be targeted for further breakdown. Two enzymes responsible for the decomposition of hydrogen peroxide are catalase and glutathione peroxidase.

**Catalase** is found in nearly all living eukaryotic organisms and exists primarily within peroxisomes as well as in the mitochondria and nucleus [33]. Catalases catalyze the breakdown of hydrogen peroxide into molecular oxygen and water:

catalase 2H_2_O_2_→O_2_ + 2H_2_O (3)

**Glutathione peroxidases (GPx)** are a class of enzymes that also break down hydrogen peroxide but do so specifically through the oxidation of a glutathione (GSH) cofactor:

GPx 2GSH + H_2_O_2_→GSSG + 2H_2_O (4)

**Paraoxonase 2 (PON2)** is a ubiquitously expressed member of the paraoxonase family of enzymes with dual functions as a lactonase and as an antioxidant enzyme. PON2 has been shown to prevent oxidation and modification of low-density lipoproteins and also counteract lipid peroxidation in the plasma membrane [34]. Additionally, PON2 interacts with the electron transport chain in the inner mitochondrial membrane to significantly reduce the production of superoxide ions [35]. The importance of PON2 as an antioxidant is demonstrated by work showing that the downregulation of PON2 significantly sensitizes cells to oxidative stress [36].

**Glutathione (GSH)** is a tripeptide comprised of three amino acids (cysteine, glutamic acid, and glycine) and is the most abundant and important low molecular weight antioxidant synthesized in both eukaryotic and prokaryotic cells. GSH plays a critical role in protecting cells from oxidative damage through direct antioxidant activity or coupled to GPx enzymatic activity [37,38]. Enzymes in the GPx family include GPx1 through 8, each with different expression patterns within the body [39]. GPx1 is the most abundant isoform and is ubiquitously expressed in the cytosol and mitochondria. GPx2 is an intestinal extracellular enzyme, while GPx3 is extracellular, and GPx4 prefers lipid peroxides. Four additional isoforms of GPx (GPx5–8) have been identified in humans but are not well studied. GPx enzymes are part of a family of critical proteins known as the phase II enzymes responsible for the conjugation of xenobiotics with peptides and sugars for detoxification.

Xenobiotic metabolism consists of phase I, phase II, and phase III enzymes involved in oxidation, conjugation/detoxification, and elimination, respectively [40,41]. Phase II enzymes are particularly important in cellular responses to oxidative stress and include GPx, glutathione S-transferase (GST), and UDP-glucuronosyltransferase (UGT). Other important antioxidant enzymes include sulfiredoxin (Srx), thioredoxin (Trx), thioredoxin reductase (TrxR), heme oxygenase 1 (HO-1), and NAD(P)H:quinone oxidoreductase 1 (NQO1). Activation of these enzymes leads to robust xenobiotic detoxification and/or antioxidant effects. Early mechanistic studies on the induction of the rat glutathione S-transferase subunit genes, *GSTA1* and *GSTA2*, led to the discovery of a specific enhancer sequence within their promoter region termed the antioxidant response element (ARE) [42]. Since then, AREs have been found in many other antioxidant genes including, among others, *NQO1* and *HMOX1* [42,43].

### 1.4. Antioxidant Response Element

**Figure 2:**
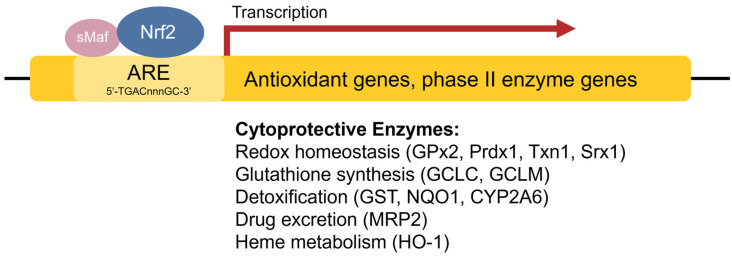
**Transcriptional regulation of antioxidant genes by the ARE and Nrf2.** Nrf2 heterodimerizes with sMaf proteins and binds to the ARE found within the promoter regions of antioxidant and phase II enzyme genes to activate their transcription.

The **antioxidant response element (ARE)** [42], also referred to as the electrophile response element (EpRE), is a cis-acting enhancer sequence found within the promoter region of many cytoprotective antioxidant and phase II enzyme genes. It has a core sequence of 5′-TGACnnnGC-3′ and participates in inducible gene expression in response to oxidative stress [42]. The ARE is also responsible for low-level basal gene expression to mitigate the ROS produced by cellular respiration. Thus, the ARE is important for redox regulation under both stressed and non-stressed conditions. Using in vivo studies in mice, Itoh et al. discovered that the induction of phase II enzymes through the ARE is mediated by a protein transcription factor called Nrf2 [44] (Figure 2). Nrf2-deficient mice showed marked reductions in the expression of the phase II enzyme GST α_1_ subunit and the antioxidant enzyme NQO1 [44], and ensuing studies demonstrated increased sensitivity to carcinogens and impaired detoxification of acetaminophen in Nrf2^−/−^ mice [45,46,47]. This illustrates the key role of Nrf2 in the activation of ARE-regulated antioxidant and phase II enzyme genes.

## 2. Keap1-Nrf2 Antioxidant Pathway

### 2.1. Keap1-Nrf2 Signalling

**Figure 3:**
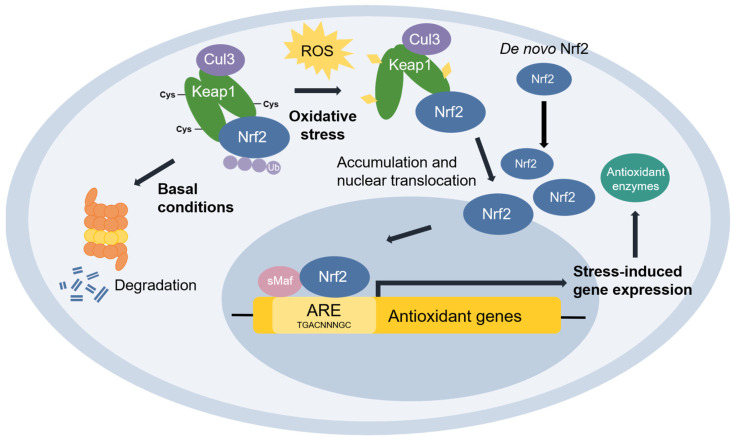
**The Keap1-Nrf2 pathway.** Under basal conditions, Keap1 is bound to Nrf2, and Nrf2 is ubiquitinated by the Cul3 E3 ubiquitin ligase for degradation by the proteasome. Upon oxidative stress, sensor cysteines in Keap1 are modified by ROS, leading to Nrf2 stabilization, accumulation, and translocation to the nucleus where Nrf2 heterodimerizes with sMaf and binds to the ARE to activate the transcription of antioxidant genes.

**Nuclear factor erythroid 2-related factor 2 (Nrf2)** [48] is the transcriptional master regulator of cellular responses against oxidative stress. Nrf2 regulates the expression of a multitude of antioxidant and phase II enzyme genes and is negatively regulated by **Kelch-like ECH-associated protein (Keap1)** [49], a substrate adaptor protein that binds to Nrf2 in the cytosol to facilitate its polyubiquitination by the Cullin 3 (Cul3) E3 ubiquitin ligase for proteasomal degradation [50,51,52]. Constitutive Nrf2 degradation allows low basal expression under non-stressed conditions. Upon oxidative stress, specific stress-sensing cysteine residues in Keap1 are modified [53,54,55], leading to a conformational change that prevents Keap1 from mediating the ubiquitination of Nrf2 by Cul3 [56]. This results in Nrf2 stabilization, accumulation, and nuclear translocation where Nrf2 heterodimerizes with sMaf proteins and binds to the ARE for the robust induction of cytoprotective genes for enzymes involved in the detoxication of ROS and other oxidants [44] (Figure 3).

### 2.2. Nuclear Factor Erythroid 2-Related Factor 2 (Nrf2)

**Figure 4:**
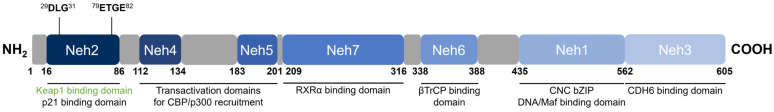
**Domain structure of human Nrf2.** Nrf2 contains seven conserved Neh domains. The Neh2 domain contains two motifs (^29^**DLG**^31^ and ^79^ETGE^82^) wherein Keap1 binds as a substrate adaptor for the Cul3-mediated ubiquitination and degradation of Nrf2.

Nrf2 [48] belongs to the cap ‘n’ collar (CNC) subfamily of basic leucine zipper (bZIP) transcription factors together with NF-E2 p45-related factors 1 and 3 (Nrf1 and Nrf3), NF-E2 p45, and transcriptional repressors BTB Domain and CNC homolog 1 and 2 (Bach1 and Bach2) [57]. Nrf2 contains seven conserved domains that are referred to as the Nrf2-ECH homology (Neh) domains, designated Neh1 through 7 (Figure 4). The key function of each domain is summarized in Table 2.

Neh1 is the DNA-binding domain that contains the CNC-bZIP region important for the association of Nrf2 with sMafs, binding to the ARE, and transcription factor activity [44,48]. The N-terminal Neh2 domain is a redox-sensitive degron that negatively regulates Nrf2 activity and contains two highly conserved ^29^**DLG**^31^ and ^79^ETGE^82^ motifs to which Keap1 binds, as well as seven lysine residues that are targets for ubiquitination by the Cul3 E3 ubiquitin ligase [49,65]. The C-terminal Neh3 domain is a transactivation domain responsible for the transcriptional activation (transactivation) of Nrf2 and has been shown to interact with chromodomain helicase DNA-binding protein 6 (CHD6) which plays a role in chromatin remodelling [59]. Neh4 and Neh5 are also transactivation domains where the binding of the CREB-binding protein (CBP) [60] or the nuclear cofactor RAC3/AIB1/SRC-3 [61] increases the rate of Nrf2 transcriptional activity. The Neh6 domain is a redox-insensitive degron that provides Keap1-independent negative Nrf2 regulation. Similar to Neh2, Neh6 contains two highly conserved ^334^DSGIS^338^ and ^373^DSAPGS^378^ motifs to which the β-transducin repeat-containing protein (βTrCP) binds, and within the DSGIS motif, a phosphorylation site for glycogen synthase kinase-3 (GSK3) that enhances βTrCP activity upon GSK3-mediated phosphorylation of Nrf2 [62,63]. Neh7 is the binding domain for retinoid X receptor α (RXRα), which upon binding impairs the recruitment of cofactors to Neh4 and Neh5 necessary for transactivation, thereby suppressing transcriptional activation [64].

### 2.3. Kelch-Like ECH-Associated Protein (Keap1)

**Figure 5:**
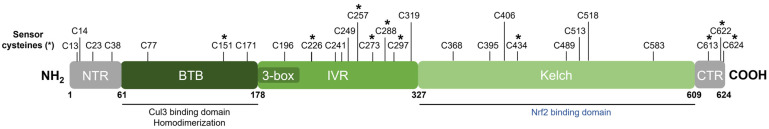
Domain structure of human Keap1. Keap1 contains three functional domains and a 3-box motif within the proximal part of the IVR domain. The location of all cysteine (C) residues in Keap1 is shown, and key stress-sensing cysteines are marked with an asterisk (*).

Keap1 [49] belongs to the BTB-Kelch family of proteins which includes about 50 members, all of which assemble with the Cul3 E3 ubiquitin ligase and RING box protein-1 (Rbx1) to form the Cullin-RING E3 ubiquitin ligases (CRLs) involved in the ubiquitination of BTB-Kelch proteins, such as Keap1 [51,66]. Cul3 assembly requires a “3-box” motif that is characteristic of BTB-Kelch proteins [67]. Accordingly, Keap1 contains three functional domains (Figure 5). The N-terminal BTB (broad complex, tramtrack, and bric à brac) domain mediates Keap1 homodimerization and contributes to its interaction with Cul3 [68]. Additional Cul3 interaction is provided by a 3-box motif found within the proximal part of the intervening region (IVR) [67]. The IVR contains key reactive cysteine residues through which Nrf2 activity is regulated, including Cys226, Cys257, Cys273, and Cys288 [53,54,55,69]. The C-terminal Kelch domain, also known as the double glycine repeat (DGR) domain, is important for Nrf2 binding [58,70].

**Figure 6:**
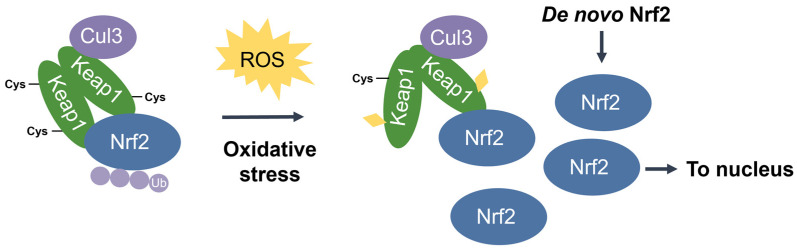
Stress-induced cysteine modification of Keap1. Under oxidative stress conditions, specific stress-sensing cysteine residues in Keap1 are modified, leading to a conformational change in Keap1 that results in Nrf2 stabilization, accumulation, and nuclear translocation for the induction of ARE-containing cytoprotective genes.

Dissociation of Nrf2 from Keap1 occurs through the oxidative modification of specific stress-sensing cysteine residues of Keap1 (Figure 6) [55]. Intriguingly, Keap1 contains a very high content of cysteines, with the 27 cysteine residues in human Keap1 accounting for approximately 4% of its total amino acid content, which is notably greater than the 2% average for the human proteome [71]. Cys273 and Cys288 are required for sensing oxidative stress under both basal and stress conditions, whereas Cys151 may be required only during oxidative stress conditions [53,54]. These three key cysteines may function independently or collaboratively depending on the class of Nrf2-inducing compounds, characterized by Yamamoto et al. [72], who also found some inducers to function independently of these three specific cysteines. Correspondingly, Cys226, Cys613, Cys622, and Cys624 are specifically involved in sensing hydrogen peroxide through a mechanism that is distinct from that used for sensing electrophilic Nrf2 inducers such that combinations of these four cysteine residues can form a disulfide bond to sense hydrogen peroxide [73]. Additional cysteine residues that respond to redox-active agents include the Cys288 alkenal sensor, the zinc sensor comprised of His225, Cys226, and Cys613, and the nitric oxide sensor comprised of a cluster of basic amino acids (His129, Lys131, Arg135, Lys150, and His154) that facilitate the *S*-nitrosylation of Cys151 within Keap1 [69].

### 2.4. Keap1-Dependent Nrf2 Regulation

As previously mentioned, Nrf2-regulated genes contain an ARE in their regulatory region and encode numerous antioxidant and phase II enzymes [44]. Transcriptional activation of the ARE is primarily dependent on Nrf2 stabilization, accumulation, and nuclear translocation through its dissociation from the cytoskeleton-associated Keap1 [49]. Thus, Nrf2 activity is tightly regulated by its interaction with Keap1.

**Figure 7:**
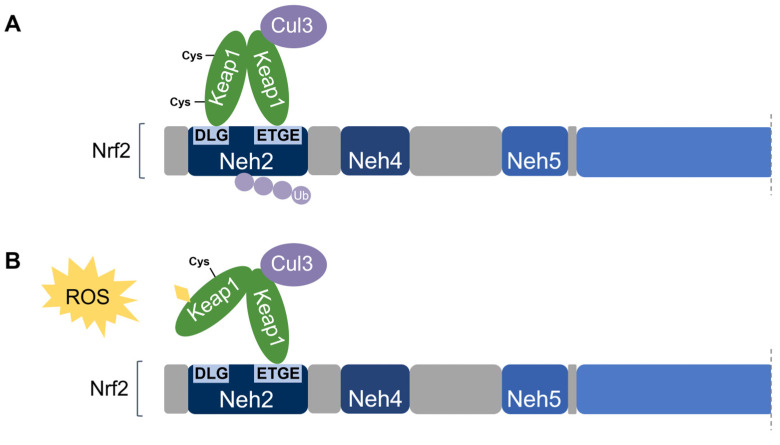
**Two-site substrate recognition model for Keap1-dependent Nrf2 regulation.** (**A**) A Keap1 homodimer binds to the Neh2 domain of Nrf2 at the DLG and ETGE motifs, allowing for the ubiquitination of Nrf2 by Cul3. (**B**) Stress-sensing cysteine residue(s) in Keap1 are modified by oxidative stress (ROS) causing a conformational change in Keap1 that impairs Nrf2-binding. Nrf2 is stabilized and no ubiquitination occurs.

Nrf2 association requires the homodimerization of Keap1 [74]. Keap1 recruits Nrf2 first through the binding of one Keap1 molecule to the high-affinity ETGE motif within the Nrf2’s Neh2 domain. Subsequent binding of the other Keap1 molecule at the low-affinity **DLG** motif locks Nrf2 in place by orienting the lysine residues within Neh2 in the correct position for ubiquitination by Cul3 and degradation by the 26S proteasome [58,65]. This is known as the two-site substrate recognition model and has been accepted as the primary mechanism of Nrf2 regulation (Figure 7).

Notably, the ETGE motif has a binding affinity that is two orders of magnitude higher than that of the DLG motif due to the presence of additional electrostatic interactions [75]. The DLG motif utilizes hydrogen bonding whereas the ETGE motif utilizes both hydrophobic interactions and hydrogen bonding [76]. Accordingly, stress-induced cysteine modifications that alter the structural conformation of Keap1 result in the prompt dissociation of Keap1 from the weak-binding DLG motif, thereby impairing Nrf2 ubiquitination. On the other hand, the Keap1-Nrf2 association may remain intact via the tight-binding ETGE motif even though ubiquitination is impaired without DLG binding [56,58]. Taken together, the DLG motif is particularly important in the Keap1-dependent degradation of Nrf2 by functioning as an “on/off switch” for Nrf2 ubiquitination. Under basal conditions, Nrf2 has a short half-life of only 10–30 min [50,77].

When Keap1-Nrf2 binding is impaired, Nrf2 may be stabilized, accumulates, and translocates to the nucleus. Within the nucleus, Nrf2 cannot bind to the ARE as a monomer and must heterodimerize with the small Maf protein (sMaf) family (MafF, MafG, MafK) for transcriptional activation of antioxidant genes [44]. Table 3 lists key examples of Nrf2-regulated genes and their associated protein function. 

For further details on the mechanisms regulating the Keap1-Nrf2 pathway, the reader is encouraged to refer to the following comprehensive reviews: [78,79,80]

### 2.5. Non-Canonical Nrf2 Regulation

Apart from its regulation by Keap1, Nrf2 is subject to further non-canonical regulation by other proteins, summarized in Table 4. Direct interaction of these proteins with either Nrf2 or Keap1 results in competitive inhibition that disrupts the Keap1-Nrf2 complex, decreases Nrf2 ubiquitination and increases Nrf2 stabilization and stress-induced ARE activation. Some of these non-canonical forms of Nrf2 regulation are discussed in further detail.

### 2.6. Nrf2-Interacting Proteins

**β-transducin repeat-containing protein (βTrCP)** participates in the negative regulation of Nrf2 via its Neh6 domain in a comparable manner to Keap1 via its Neh2 domain. βTrCP interacts with Neh6 at two conserved sites, ^334^DSGIS^338^ and ^373^DSAPGS^378^, and acts as a substrate receptor for degradation by the Skp1-Cul1-Rbx1/Roc1 E3 ubiquitin ligase complex [62,63]. Deletion of either motif results in the loss of βTrCP-mediated ubiquitination [62]. Additionally, the DSGIS motif in Neh6 overlaps with a phosphorylation site for GSK3, wherein phosphorylation of Nrf2 at this motif by GSK3 enhances βTrCP activity [62,63]. Accordingly, when Keap1 activity is impaired in Keap1^−/−^ mouse embryonic fibroblasts or in an Nrf2 ETGE deletion mutant that cannot bind to Keap1, treatment with GSK3 inhibitors leads to impaired βTrCP-regulation and results in Nrf2 stabilization and accumulation [62]. On the other hand, activation of GSK3 in Keap1^−/−^ mouse embryonic fibroblasts or human lung A549 cells reduces Nrf2 protein levels and mRNA levels for Nrf2-regulated enzymes [63].

**Retinoid X receptor alpha (RXRα)** is involved in numerous developmental and physiological pathways and in mediating the biological effects of retinoids [94]. RXRα directly interacts with the Neh7 domain of Nrf2, which impairs the recruitment of cofactors to Neh4 and Neh5 required for transactivation [64]. Accordingly, RNAi-mediated knockout of RXRα increases the induction of Nrf2-regulated antioxidant gene expression, and overexpression of RXRα in non-small cell lung cancer A549 cells leads to Nrf2 downregulation and increases sensitivity to therapeutic drugs [64].

**p21 (or p21^CIP1/WAF1^)** is a p53-regulated cyclin-dependent kinase inhibitor involved in inhibiting the activity of cyclin/cyclin-dependent kinase (Cdk) complexes for the negative regulation of cell cycle progression [24]. The ^154^**KRR**^156^ motif within p21 directly binds to the DLG and ETGE motifs in Nrf2, thereby competing with Keap1 for Nrf2 binding [26]; but instead of Nrf2 degradation, p21-Nrf2 binding leads to Nrf2 stabilization and increased response to oxidative stress [26]. Accordingly, p21^−/−^ mice show reduced levels of Nrf2 protein and Nrf2 target genes [26]. Importantly, p21-dependent protection from oxidative stress requires Nrf2, as colorectal cancer HCT116 cells overexpressing p21 demonstrate enhanced survival in response to hydrogen peroxide in Nrf2^+/+^ but not Nrf2^−/−^ cells [26]. 

**Protein deglycase DJ-1 (DJ-1)** (also known as Park7) is a redox-dependent molecular chaperone that mediates protein folding and prevents the misfolding and inclusion formation of neuronal proteins such as α-Synuclein [95]. DJ-1 inhibits Keap1-mediated Nrf2 degradation by competitively binding to Nrf2 [81]. In both primary human cells and mice, loss of DJ-1 leads to deficits in the expression of Nrf2-mediated stress response enzymes, particularly the detoxification enzyme NQO1, suggesting that DJ-1 is required for Nrf2 stability and Nrf2-mediated transcription [81]. Notably, mutations in the DJ-1 gene are associated with early-onset Parkinson’s disease (PD) [96], suggesting the role of impaired oxidative stress regulation in neurodegenerative diseases, such as PD. 

**Breast cancer type 1 susceptibility protein (BRCA1)** is a tumour suppressor protein primarily responsible for DNA damage repair in cells of the breast and other tissue [97]. The BRCT domain of BRCA1 interacts with the ETGE motif in the Neh2 domain of Nrf2, which inhibits Keap1-mediated ubiquitination and increases the response to oxidative stress [82,83]. Expression of BRCA1 in neurons confers protection from ischemia/reperfusion injury through activation of the Nrf2-mediated antioxidant pathway [83], and BRCA1^−/−^ mouse primary mammary epithelial cells demonstrate low expression of Nrf2 target genes and increased ROS levels associated with decreased survival [82]. Intriguingly, BRCA1 contains an ARE sequence in its promoter region and is thereby regulated by Nrf2, creating a positive feedback loop [98].

### 2.7. Keap1-Interacting Proteins

**p62** (also known as sequestosome-1, SQSTM1) is a stress-inducible scaffold protein involved in numerous signalling pathways, including the targeting of proteins for selective autophagy [92,93]. In 2010, five independent groups discovered the interaction between p62 and Keap1 [77,78,79,80,81]. This interaction is mediated by a 349DPSTGE354 motif in p62’s Keap1-interacting region (KIR) that resembles the ETGE motif in the Keap1-binding domain of Nrf2 [79,80,81]. p62 sequesters Keap1 into inclusion bodies for autophagy-mediated degradation, thereby disrupting the Keap1-Nrf2 interaction and inhibiting Nrf2 ubiquitination. Additionally, the binding affinity between p62 and Keap1 is significantly increased when Ser351 in p62 is phosphorylated, leading to increased Nrf2 transcriptional activity [94]. Notably, p62 contains ARE sequences in its promoter and is thereby regulated by Nrf2, indicating a positive feedback loop [79].

Prothymosin α (ProTα/PTMA) is a small, highly charged protein involved in cell proliferation and survival through chromatin remodelling and anti-apoptotic activity [95,96]). ProTα interacts with the Kelch domain of Keap1 and shuttles it into the nucleus, thereby preventing its association with Nrf2 [82]. The 38NANEENGE45 motif in ProTα is required for its interaction with the Kelch domain [97]. HeLa cells overexpressing ProTα show increased Nrf2-mediated HMOX1 gene expression; however, overexpression of a mutant variant of ProTα that impairs Keap1-binding fails to upregulate HMOX1 [82], thereby demonstrating the role of ProTα in the expression of certain antioxidant genes.

**Dipeptidyl-peptidase 3 (DPP3)** participates in the cleavage and degradation of bioactive peptides generated by the proteasome during protein degradation [98,99]. DPP3, which contains a 480ETGE483 motif, interacts with Keap1 by binding to its Kelch domain, thereby inhibiting the Keap1-Nrf2 interaction [83]. Estrogen receptor-positive MCF7 breast cancer cells demonstrate overexpression of DPP3 which is associated with increased Nrf2 gene expression and poor prognosis [100].

**WTX** is a tumour suppressor and regulator in the canonical Wnt signalling pathway, which mediates critical aspects of embryonic development by promoting the ubiquitination and degradation of β-catenin [101,102]. WTX is also involved in oxidative stress regulation through its competitive binding to the Keap1, which inhibits Nrf2 ubiquitination [84]. siRNA knockdown of WTX in HEK293T cells reduces the activation of Nrf2 target genes in response to tBHQ, a potent Nrf2-activating compound [84]. WTX contains a 286SPETGE291 motif that is similar to the ETGE motif in Nrf2, which allows for interaction with the Kelch domain in Keap1; however, this interaction requires the phosphorylation of Ser286 to attain a sufficient binding affinity between the two proteins [84].

**Partner and localizer of BRCA2 (PALB2)** (also known as Fanconi anemia complementation group N, FANCN), is a protein that co-localizes with the breast cancer 2 early onset protein (BRCA2) to regulate its stabilization, nuclear localization, and involvement in DNA repair [103]. siRNA knockdown of PALB2 in bone-derived U2OS cells results in reduced Nrf2 activity and increased ROS levels [103]. Like the WTX protein, PALB2 contains a 91ETGE94 motif that permits its interaction with Keap1 through binding to the Kelch domain [85].

**KPNA6** (also known as importin α7) is a nucleocytoplasmic transport adaptor involved in the nuclear import of proteins. Keap1 has been shown to shuttle between the nucleus and cytoplasm via KPNA6 which interacts with the Kelch domain of Keap1. Within the nucleus, Keap1 binds to Nrf2 to facilitate its nuclear export and subsequent ubiquitination in the cytosol, thus allowing for attenuation of Nrf2 activity during the postinduction phase [86]. Knockdown of KPNA6 impairs Keap1 nuclear shuttling and attenuates the Keap1-mediated ubiquitination of Nrf2, whereas overexpression of KPNA6 facilitates Keap1 nuclear import and inhibits Nrf2 signalling [86].

### 2.8. Other Mechanisms of Nrf2 Regulation

The transcriptional activity of Nrf2 may also be inhibited by Bach1, a protein in the same CNC-bZIP family as Nrf2 that functions as a transcriptional repressor. Bach1 competes with Nrf2 in the nucleus for heterodimerization with the sMaf proteins which are required for Nrf2/ARE binding [99]. Other forms of Nrf2 regulation include phosphorylation of Nrf2 at Ser40 by protein kinase C (PKC), which impairs Keap1 binding [100], and phosphorylation of Nrf2 by the MAPK/ERK pathway, which increases Nrf2 stability [50]. 

Finally, phosphoglycerate mutase family member 5 (PGAM5) is a protein phosphatase with functions in mitochondrial homeostasis and mitophagy [101]. Interestingly, PGAM5 can recruit both Keap1 and Nrf2 to the outer mitochondrial membrane by binding to one molecule of a Keap1 dimer while simultaneously binding Nrf2 to form a ternary Keap1-PGAM5-Nrf2 complex [102,103]. Interestingly, this results in the stress-induced Keap1-mediated ubiquitination and degradation of not Nrf2, but of mitochondrial Rho GTPase 2 (Miro2), a mitochondrial GTPase involved in mitochondrial motility [104]. This demonstrates that Nrf2 function is not limited to stress-induced gene transcription but highlights its involvement in other cellular processes.

## 3. Nrf2 in Human Disease

Due to its crucial role in oxidative stress regulation and additional roles in many other cellular processes, aberrant Nrf2 expression has been associated with numerous pathologies. Some major human diseases, including cardiovascular disease, diabetes, neurodegenerative disease, psychiatric disorders, and cancer are briefly discussed in the following section.

### 3.1. Neurodegenerative Disease

The link between oxidative stress and the pathogenesis of neurodegenerative diseases is well-established [22,105]. The brain consumes 20% of the body’s oxygen relative to its small mass (~2% of total body mass) and is particularly susceptible to oxidative damage due to its high rate of metabolic activity, high rate of oxygen metabolite production, relatively low levels of antioxidants, low capacity for repair, and high composition of lipids which are prone to peroxidation and oxidative modification by ROS [106,107]. Damaged mitochondria and activated microglia are major sources of ROS in the brain [105]. Oxidative damage has been implicated in all major neurodegenerative diseases including Alzheimer’s disease (AD) [108,109], Parkinson’s disease (PD) [110,111], Huntington’s disease (HD) [112,113], amyotrophic lateral sclerosis (ALS) [114], and multiple sclerosis (MS) [115]. Except for MS, all of these diseases are characterized by the loss and/or deterioration of neurons in a specific brain region due to hallmark protein misfolding and inclusion formation [116].

High levels of oxidative damage exceeding that found in healthy, aging brains, have been observed in the post-mortem brain tissues of patients with neurodegenerative diseases [105], suggesting that oxidative stress plays a role in the formation and/or aggravation of these hallmark protein inclusions. Nrf2 is activated in response to oxidative stress but may be impaired or insufficient in neurodegenerative diseases. Significantly reduced levels of nuclear Nrf2 have been observed in the brain regions of AD patients [117]. Conversely, while Nrf2 nuclear localization is observed in PD patient samples, the response may be insufficient to prevent neuronal cell death [118]. Additionally, studies have reported the protective role of Nrf2 in neurodegenerative diseases [119,120]. For example, Nrf2 activation in astrocytes confers protection against neurodegeneration in mouse models of ALS [121], and Nrf2 deficiency results in increased sensitivity to MPTP-induced PD-like lesions in mice which is improved by Nrf2 overexpression in astrocytes [122]. Nrf2 inducers have been shown to have protective effects in the development of neurogenerative disease-associated brain lesions [120]. The current status of Nrf2 in neurodegenerative disease is comprehensively reviewed by Zgorzynska et al. (2021) [123], Cuadrado (2022) [124], George et al. (2022) [125].

### 3.2. Neuropsychiatric Disorders

Oxidative stress has been implicated as a pathogenic mechanism underlying psychiatric disorders and their associated neurodegenerative changes. This includes, but is not limited to, schizophrenia, bipolar disorder, depression, anxiety, autism, and attention deficit hyperactivity disorder (ADHD) [126,127]. The pathophysiological mechanisms vary between each disorder and remain largely underexplored; however, increasing preclinical and clinical evidence suggests that higher levels of oxidative stress, alterations in the Keap1-Nrf2 pathway, and Nrf2-associated inflammation are involved in the pathogenesis of these psychiatric disorders [128,129,130,131,132].

Evidence of oxidative disturbances in the aforementioned disorders has been demonstrated in both human and animal studies examining oxidative markers, the antioxidant properties of antidepressants, and antioxidant therapies, summarized in comprehensive reviews by Ng et al. [126] and Smaga et al. [127]. Oxidative stress in psychiatric disorders could result from the overproduction of ROS, impairments in Keap1-Nrf2 signalling, or both due to causes related to psychological stress, physiological impairments, inflammation, and genetic polymorphisms of antioxidant enzymes that are beyond the scope of this review [126,127]. Notably, however, changes in Keap1-Nrf2 signalling have been observed in postmortem brain tissue from patients with major depressive disorder and bipolar disorder which showed marked reductions in protein levels of Keap1 and Nrf2 in the parietal cortex compared to the control group [133]. Alterations in Keap1 and Nrf2 have also been observed in rodent models of depression. Mice subjected to chronic social defeat stress (CSDS) develop depression-like symptoms [134,135] and express lower protein levels of Keap1 and Nrf2 in area CA3 in the hippocampus, dentate gyrus, and prefrontal cortex compared to healthy controls [133], indicating that reduced levels of Keap1 and Nrf2 in these brain areas may contribute to the depression-like phenotypes following CSDS. Moreover, CSDS mice demonstrate higher levels of inflammatory cytokines compared to controls [136]. Growing evidence suggests that pro-inflammatory cytokines contribute to the pathogenesis of psychiatric diseases such as depression [137,138], and given the pivotal role of Nrf2 in inflammatory processes (discussed in Section 3.6), Nrf2 could also play a role in depressive disorders through its anti-inflammatory mechanisms [139]. Importantly, antioxidants have shown promising therapeutic benefits in the treatment of psychiatric disorders and are subject to further investigation [126,127,140]. For additional information of Nrf2 in neuropsychiatric disorders, refer to the reviews by Hashimoto (2018) [132] and Morris et al. (2021) [141].

### 3.3. Cardiovascular Disease

Cardiovascular disease is a multifaceted disease with a variety of risk factors including hypercholesterolemia, hypertension, and atherosclerosis [142]. Oxidative stress may play a role in the development of vascular complications that promote cardiovascular disease by contributing to the pathogenesis of hypertension [143,144] and atherosclerosis [145]. The endothelial isoform of nitric oxide synthase (eNOS) is responsible for the biosynthesis of NO in endothelial cells which mediates vascular relaxation [146]. The uncoupling of eNOS under pathogenic conditions (e.g., hypertension, atherosclerosis, or diabetes) results in both impaired NO production and increased superoxide production, which leads to hypertension and blood vessel damage, respectively [147]. Additionally, increased oxidative stress has been found to promote the conversion of harmful low-density lipoprotein (LDL) cholesterol to the more atherogenic oxidized LDL form (oxLDL) [148,149]. Nrf2 has been shown to protect cardiomyocytes from ROS-induced damage through the expression of antioxidant enzymes [150,151] while lack of Nrf2 promotes aggravation of vessel lesions towards atherosclerosis [145]. Nrf2 is thus a critical regulator of cardiovascular homeostasis with implications for the development of cardiovascular disease. For additional readings on this topic, refer to da Costa et al. (2019) [152], Gutiérrez [153]-Cuevas et al. (2022), and Wu et al. (2022) [154].

### 3.4. Diabetes and Diabetic Complications

Diabetes mellitus is characterized by hyperglycemia, which induces the excess generation of ROS, leading to oxidative stress (Inoguchi et al., 2000). Chronic oxidative stress is a major contributor to various diabetes-specific complications, particularly diabetic nephropathy, and cardiomyopathy, as well as neuropathy, retinopathy, and accelerated atherosclerotic disease (Cai & Kang, 2001; Vincent et al., 2004; Madamanchi et al., 2005; Kowluru & Chan, 2007; Giacco & Brownlee, 2010; Negi et al., 2011; Liu et al., 2014; Calderon et al., 2017). As a transcription factor, Nrf2 plays a crucial role in protecting cells and tissues against diabetes-induced oxidative damage (He et al., 2009; Jiang et al., 2010b). Accordingly, upregulated levels of Nrf2 have been observed in the kidneys and hearts of diabetic patients. In diabetic nephropathy patients, Jiang et al. found a significant increase in Nrf2 expression levels upon immunohistochemical analysis of the glomeruli of diabetic patients compared to nondiabetic controls. Using human mesangial cells (HRMCs), the authors also demonstrated that high glucose levels increase the nuclear translocation of Nrf2 which is associated with increases in the Nrf2 target genes NQO1, HO-1, and GST (Jiang et al., 2010b). The importance of Nrf2 in preventing high glucose-induced oxidative damage is further demonstrated in several studies comparing diabetic Nrf2-KO mice with wild-type mice. Following treatment with streptozotocin (STZ) to induce diabetes, diabetic Nrf2-KO mice demonstrated greater deterioration of renal function and higher renal ROS production compared to their wild-type counterparts (Yoh et al., 2008; Jiang et al., 2010b). Similarly, in primary cardiomyocytes, high glucose concentrations induce ROS production that is associated with increased mRNA and protein expression levels of Nrf2 and several of its target genes; however, in Nrf2 KO cells, ROS levels are significantly higher at baseline and markedly higher under high glucose conditions and associated with significant levels of apoptosis (He et al., 2009). High glucose-induced Nrf2 activation has also been demonstrated in endothelial cells (Ungvari et al., 2011a) and vascular smooth muscle cells (Hur et al., 2010). Consequently, Nrf2 activators have been investigated as a therapy and in the prevention of diabetic complications by combating oxidant-induced damage. For example, Nrf2 activation by the phytochemical sulforaphane is found to reverse and prevent the biochemical dysfunction in endothelial cells induced by hyperglycemia (Xue et al., 2008), among others [155]. Taken together, Nrf2 is important in mitigating the ROS-induced damage caused by diabetic hyperglycemia, and induction of the Nrf2 pathway could be a promising therapeutic strategy for preventing diabetes-associated complications. Refer to Uruno et al. (2015) [156] and Tanase et al. (2022) [157] for additional details on Nrf2 in diabetes.

### 3.5. Cancer

**Figure 8:**
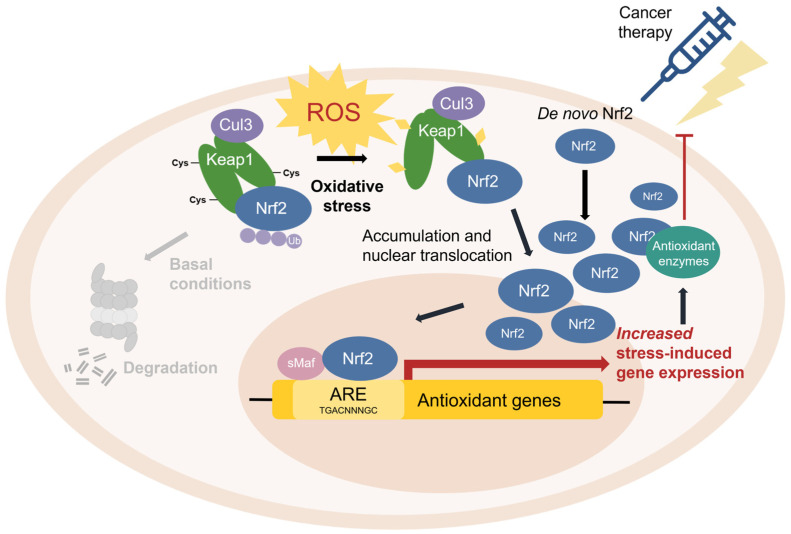
The aberrant Keap1-Nrf2 pathway in cancer. Some mutations are associated with Nrf2 hyperactivation, which protects cancer cells from ROS and chemotherapeutic agents by increased antioxidant activity.

Most cancers show elevated levels of ROS and oxidative stress, which can cause DNA damage, impair protein function, and alter mechanisms of cellular proliferation to promote tumorigenesis [154]. This is particularly true for cancers of the skin and lung, as both organs are directly exposed to additional sources of environmental ROS which contribute to cancer initiation and aggravation [158,159]. Traditionally, Nrf2 has been considered a tumour suppressor that confers protection against ROS and cancer progression. For instance, mice deficient in Nrf2 are prone to chemical-induced toxicity and tumorigenesis [160]. However, despite its beneficial role in cellular protection and cancer prevention, Nrf2 also has a harmful “dark side” in cancer [156]. Some somatic mutations give rise to hyperactive Nrf2, which causes enhanced antioxidant capacity and confers protection of cancer cells from ROS and cancer therapy, thereby leading to cancer cell growth and proliferation, cancer progression, and cancer therapy resistance (e.g., chemoresistance) (Figure 8) [161,162,163,164,165,166,167,168,169,170,171].

**Figure 9:**
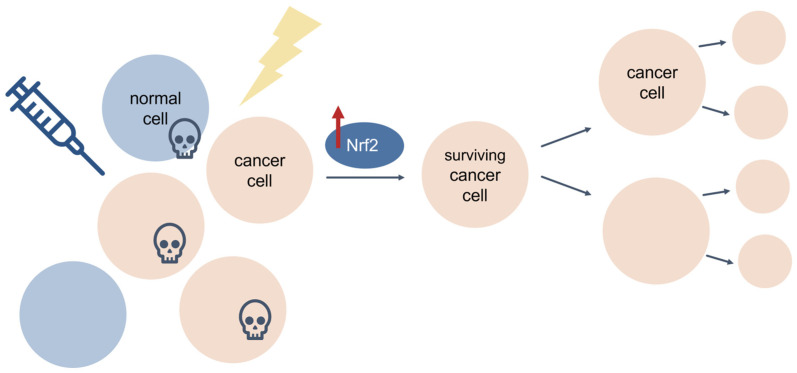
Nrf2 protects cancer cells from cancer therapy. Cancer cells hijack the Nrf2 pathway to confer protection against cancer therapies such as chemotherapy and radiation. Cancer cells that survive therapy develop resistance and proliferate, leading to chemoresistance and cancer progression.

Constitutive Nrf2 hyperactivation is common in cancer [172], and numerous studies have revealed aberrant Nrf2 expression and poor prognosis in a wide range of cancers, including, among others, lung, esophageal, breast, bladder, liver, prostate, and colorectal carcinomas [161,162,169,170,171,173,174], most of which have been attributed to loss-of-function mutations in the *KEAP1* gene and/or gain-of-function mutations in the *NFE2L2* gene encoding Nrf2 [162,163,164,166,167]. Mutations in *KEAP1* were first discovered in human lung adenocarcinoma cell lines, wherein a glycine-to-cysteine substitution within the Nrf2-binding domain of Keap1 reduces its affinity for Nrf2, resulting in loss of canonical Nrf2 regulation and constitutive Nrf2 hyperactivation [163]. Similarly, mutations within the Keap1-binding domain of Nrf2 impair Keap1 recognition, allowing Nrf2 to escape Keap1-mediated degradation and accumulate at high levels in cancer cells [162]. Genomic characterization of squamous cell lung cancers showed significant alterations in the Nrf2 pathway in 34% of all tumour specimens examined [175]. Mutation frequencies vary greatly across different cancer types, but interestingly, some cancers show high rates of Nrf2 pathway alterations but low rates of *KEAP1* or *NFE2L2* mutations. This suggests that aberrant Nrf2 regulation in cancer may be due to Keap1-independent Nrf2 regulatory pathways, or impaired Keap1-Nrf2 interactions at the protein level. While it is unlikely that these mutations cause cancer, Nrf2 mutations may enhance the growth and development of existing cancer cells by conferring enhanced antioxidant abilities to compensate for the hostile microenvironment of a rapidly dividing cancer cell where ROS is abundant and oxidative stress is high [176,177]. Nrf2 hyperactivation creates an environment that protects normal but also malignant cells from oxidative stress and cancer therapy. The resultant upregulation of Nrf2-mediated antioxidant proteins renders cancer cells resistant to chemotherapeutic drugs (e.g., cisplatin, 5-fluorouracil, docetaxel, and bortezomib) and radiotherapy [47,161,165,178,179,180,181,182,183,184]. Thus, cancer cells appear to hijack the Nrf2 antioxidant pathway to evolve protection against chemotherapeutics to promote chemoresistance and tumorigenesis (Figure 9). With this information, researchers can target the Keap1-Nrf2 pathway as an anticancer strategy against cells that develop chemoresistance (further discussed in Section 3.8). 

Many great reviews have been written on the topic of oxidative stress and Nrf2 in cancer. The reader is encouraged to refer to the following reviews for additional details. For oxidative stress in cancer, refer to Hayes et al. (2020) [185]. For further reading on the role of Nrf2 in cancer, refer to Taguchi et al. (2017) [177], Rojo de la Vega et al. (2018) [176], and Panda et al. (2022) [186]. For additional details on Nrf2 as a therapeutic target in cancer, refer to Taguchi et al. (2020) [187] and Sivinski et al. (2021) [188].

### 3.6. Inflammation

Inflammation is a biological defence mechanism that is triggered in response to harmful insults such as pathogens, toxins, injury, and damaged cells. Through cytokine production and the recruitment of inflammatory cells, inflammation aims to eliminate the insult, limit its spread, and clear the area for healing and repair [189,190]. Nrf2 plays a role in regulating the anti-inflammatory response through redox control and activation of ARE-mediated anti-inflammatory genes, including the expression of the antioxidant genes *NQO1*, *HO-1*, and *PRX1*, all of which exhibit anti-inflammatory effects [191,192,193,194]. The anti-inflammatory role of Nrf2 also includes Nrf2-mediated inhibition of the pro-inflammatory NF-_Κ_B pathway and inhibition of expression of pro-inflammatory cytokines [195,196,197]. Of note, the expression of pro-inflammatory cytokine genes in M1 macrophages is inhibited by Nrf2-ARE binding [198]; however, Nrf2 has also been found to block the transcriptional upregulation of pro-inflammatory cytokine genes including interleukin 6 (IL-6) and interleukin 1 beta (IL-1β) in an ARE-independent manner through direct binding to the proximity of pro-inflammatory genes to inhibit RNA polymerase II recruitment, suggesting that Nrf2’s role in inflammation is not limited to just oxidative stress control [198]. Nrf2 plays numerous additional roles in inflammation that are summarized in review articles published by Ahmed et al. (2017) [199] and Saha et al. (2020) [200].

### 3.7. Aging

Aging is not a disease per se, but a predominant risk factor for the development of disease. Progressive and irreversible oxidative damage accumulates with age and diminishes critical aspects of cell physiology [57,201,202]. For example, aging is associated with impaired activity of the proteasome and mitochondrial Lon proteases [203,204,205] and reduced capacity for macromolecule repair [201,202]. The “oxidative damage theory of aging” [206] thus postulates that: (1) age-related functional losses are caused by the gradual accumulation of ROS and general oxidative damage to macromolecules, and that (2) ROS reduction and oxidative damage repair attenuate the rate of aging and increases lifespan. In line with this hypothesis, Nrf2 signaling has been found to decrease with age [207] in a variety of model organisms including flies [208], mice [209], non-human primates [210,211], and humans [207,212,213,214]. 

Notably, experimental amplification of Nrf2-regulated antioxidant genes has been found to increase resistance to oxidative stress in some aged model systems but not others [207], indicating that increased steady-state levels of ROS and oxidized macromolecules may not be the only contributor to age-related functional losses. The alternative “redox stress hypothesis” [215] instead proposes that impairments in physiologic function are due to an age-related “pro-oxidizing shift” in the redox state of cells that results in the over-oxidation of redox-sensitive thiol groups within the cysteine residues of proteins, resulting in the impairment of cellular signaling pathways. Much evidence suggests that oxidative damage to proteins is associated with aging and is linked to protein misfolding [216,217]. For additional details on oxidative stress in aging and disease, refer to and Liguori et al. (2018) [21] and Tan et al. (2018) [218]. Additionally, Schmidlin et al. (2019) provide a comprehensive review on the role of Nrf2 in aging and disease [219].

### 3.8. Nrf2 as a Therapeutic Target

The importance of Nrf2 in the protection against human diseases is well established and much research has explored the use of Nrf2 activators in the treatment of disease [220,221,222,223]. Examples include dimethyl fumarate, which has been approved for the treatment of multiple sclerosis [224], sulforaphane [225], and numerous others currently in clinical trials [226,227]. While some Nrf2 activators have shown promise, elevated levels of Nrf2 can have negative effects, as observed in chemotherapy-resistant cancer cells. Research has thus also explored the use of Nrf2 inhibitors as adjuvants to cancer therapy [226,228]. For example, brusatol has been shown to enhance the efficacy of chemotherapy by inhibiting Nrf2 [229,230]. Among these Nrf2 modulators, there are natural compounds that target and significantly inhibit the Keap1-Nrf2 pathway, most of which are safe (including dietary phytochemicals) and have shown promise against chemoresistant cancer cells [223,231]. It should be mentioned that Nrf2 upregulation has also been observed for non-cancerous treatment applications when the drug is revealed to be toxic, such as treatment with bardoxolone methyl in patients with type 2 diabetes mellitus and stage 4 chronic kidney disease [232]. This suggests that upregulation of the antioxidant pathway is an adaptive mechanism against drug toxicity for both cancerous and non-cancerous treatment scenarios. Thus, targeting Nrf2 for the treatment of human disease has shown promise, and an increased understanding of the delicate balance between Nrf2’s protective and deleterious effects will contribute to its value as a therapeutic target.

## 4. Conclusions

Given its multi-faceted roles in normal cell physiology and disease, Nrf2 has been extensively studied since its discovery in 1994 [48]. Nrf2 regulates a wide array of antioxidant genes, and in turn, Nrf2 activity is highly regulated by its numerous protein interaction partners, some of which are briefly discussed in this review. This multi-faceted nature of Nrf2 also results in its implication in a wide range of human diseases, which are also briefly discussed. Taken together, this review provides a general overview of oxidative stress and Nrf2, including basic mechanisms and implications in human disease. For more detailed discussions, the reader is encouraged to peruse the rich body of Nrf2 literature that is available.

## Figures and Tables

**Table 1:** Major Reactive Oxygen and Reactive Nitrogen Species.

Molecule Type	Radical Status	Name	Symbol
ROS	Radical	Molecular oxygen	O_2_••
		Superoxide	O_2_•^−^
		Hydroxyl	•OH
		Alkoxyl	RO•
		Peroxyl	ROO•
		Hydroperoxyl	HO_2_•
			
	Non-radical	Hydrogen peroxide	H_2_O_2_
		Peroxide	ROOR
		Singlet oxygen	O_2_
		Ozone	O_3_
		Hydroxyl ion	OH^−^
		Peroxynitrite	ONOO^−^
RNS	Radical	Nitric oxide	•NO
		Nitrogen dioxide	•NO_2_
			
	Non-radical	Peroxynitrite	ONOO^−^
		Alkyl peroxynitrite	ROONO
		Nitronium cation	NO^2+^
		Nitroxyl cation	NO^+^
		Nitroxyl anion	NO^−^
		Nitrogen oxides	N_x_O_x_

**Table 2:** Summary of functional domains of Nrf2 and their key binding proteins.

Domain	Key Associated Function	Binds to	Ref.
Neh1	DNA-binding via the ARE; dimerization with sMaf proteins	sMaf, ARE	[44,48]
Neh2	Keap1-binding for negative regulation	Keap1	[49,58]
Neh3	Transactivation	CHD6	[59]
Neh4, Neh5	Transactivation	CBP	[60,61]
Neh6	βTrCP-binding for negative regulation	βTrCP	[62,63]
Neh7	RXRα-binding for suppressed transactivation	RXRα	[64]

**Table 3:** Examples of cytoprotective genes regulated by Nrf2.

Primary Role	Gene	Protein	Function
**Redox homeostasis**	*GPX2*	Glutathione peroxidase 2 (GPx2)	Reduces hydrogen peroxide and lipid hydroperoxides at the expense of glutathione
	*PRDX1*	Peroxiredoxin 1 (Prdx1)	Reduces hydrogen peroxide and alkyl hydroperoxides
	*TXN1*	Thioredoxin 1 (Trx1)	Reduces oxidized protein thiols
	*SRXN1*	Sulfiredoxin 1 (Srx1)	Contributes to the thioredoxin system by reducing sulfinic acid to thiols
			
**Glutathione biosynthesis**	*GCLC*	Glutamate-cysteine ligase catalytic subunit (GCLC)	The first rate-limiting enzyme of glutathione synthesis (heavy subunit)
	*GCLM*	Glutamate-cysteine ligase modifier subunit (GCLM)	The first rate-limiting enzyme of glutathione synthesis (light subunit)
			
**Detoxification**	*GST*	Glutathione S-transferase (GST)	Catalyzes the conjugation of glutathione to electrophilic compounds
	*NQO1*	NAD(P)H:quinone oxidoreductase-1 (NQO1)	Reduces quinone to hydroquinone
	*CYP2A6*	Cytochrome P450 2A6 (CYP2A6)	Involved in the hydroxylation of some anti-cancer drugs
			
**Drug Excretion**	*ABCC2*	Multidrug resistance protein 2 (MRP2)	Mediates hepatobiliary excretion; implicated in multidrug resistance
			
**Heme metabolism**	*HMOX1*	Heme oxygenase 1 (HO-1)	Cleaves heme to form biliverdin during heme catabolism

**Table 4:** Non-canonical Nrf2 regulation by direct protein interaction.

	Interacting Protein	Known Interaction Motif(s)	Nrf2 Domain	+ or −Nrf2 Regulation	Ref.
**Nrf2**	βTrCP	^334^DSGIS^338^ (Nrf2) ^373^DSAPGS^378^ (Nrf2)	Neh6	**−**; Nrf2 degradation	[62,63]
	RXRα	^209^ETT…NGP^316^ (Nrf2)	Neh7	**−**; ↓ transactivation	[64]
	p21	^29^**DLG**^31^ (Nrf2) ^79^ETGE^82^ (Nrf2) ^154^**KRR**^156^ (p21)	Neh2	+; Nrf2 stabilization	[26]
	DJ-1	Currently unknown	---	+; Nrf2 stabilization	[81]
	BRCA1	^79^ETGE^82^ (Nrf2) BRCT domain ^(1591–1784)^ (BRCA1)	Neh2	+; Nrf2 stabilization	[82,83]
	**Interacting Protein**	**Interaction Motif(s)**	**Keap1 Domain**	**+ or −Nrf2 Regulation**	**Ref.**
**Keap1**	p62/SQSTM1	^349^DPSTGE^354^ (p62)	Kelch	+; Keap1 inhibition	[84,85,86,87,88]
	ProTα/PTMA	^38^NANEENGE^45^ (ProTα)	Kelch	+; Keap1 inhibition	[89]
	DPP3	^480^**ETGE**^483^ (DPP3)	Kelch	**+**; Keap1 inhibition	[90]
	WTX	^286^**SPETGE**^291^ (WTX)	Kelch	**+**; Keap1 inhibition	[91]
	PALB2/FANCN	^91^**ETGE**^94^ (PALB2)	BTB	+; Keap1 inhibition	[92]
	KPNA6/Importin α7	ARM domain ^(108–563)^ (KPNA6)	Kelch	−; Nrf2 degradation	[93]

## Data Availability

Not applicable.
